# Great Surabaya Cadres play an important role in achieving health programs in Surabaya, Indonesia

**DOI:** 10.11604/pamj.2024.49.58.45183

**Published:** 2024-10-29

**Authors:** Titi Maharrani, Evi Yunita Nugrahini, Domas Nurchandra Pramudianti, Mohammad Dwinanda Junaedi

**Affiliations:** 1 Department of Midwifery, Poltekkes Kemenkes Surabaya, Surabaya, Indonesia,; 2 Faculty of Medicine, Nahdlatul Ulama University Surabaya, Surabaya, Indonesia

**Keywords:** Great Surabaya Cadres, health programs, community empowerment, community health centers

## Abstract

Surabaya as a metropolitan city is required to be able to provide health services that can reach the entire community. Therefore, the Surabaya city government empowers the community to play an active role in the health sector by forming the Great Surabaya Cadres (in Indonesian, KSH = Kader Surabaya Hebat). Great Surabaya Cadres have three tasks, which include data collection and updating of residents' health data; collaborating with health workers at the health center in implementing the integrated development place program (in Indonesian, Posbindu), integrated service place for toddlers (in Indonesian, Posyandu balita), and integrated service place for the elderly (in Indonesian, Posyandu lansia) and implementing the “Jumantik” program as an effort to control dengue fever. It can be concluded that Great Surabaya Cadres have an important role in supporting the achievement of health programs initiated by the government.

## Opinion

The health condition of a city is a reflection of the interaction of various factors including the physical, social, and economic environment. A healthy city is a city that is able to provide adequate health services for all citizens [1]. Surabaya is one of the metropolitan cities in Indonesia with a population of approximately 3,009,286 people and has 63 Community Health Centers (*Puskesmas*) spread across each sub-district. As an effort to provide good health services, the Surabaya city government empowers the community by forming the Great Surabaya Cadres (In Indonesian, KSH = *Kader Surabaya Hebat*) in each sub-district [2]. The Great Surabaya Cadres is someone who is part of the community and meets certain requirements [3] such as having an identity card, residing in Surabaya, having good physical and mental health, maximum of 60 years of age, a minimum education of junior high school and able to operate simple applications. The Great Surabaya Cadres will be given training according to the field of expertise needed in running the program.

The Great Surabaya Cadre in the health sector is under the supervision of the local Community Health Center. Each cadre has a different work area and is required to report the results of their activities to the Community Health Center. In general, there are three tasks of the Great Surabaya Cadres. Initially, they had collected and updated residents' health conditions. The Great Surabaya Cadres collect data and update data on residents' health conditions through an application, namely *"Sayang Warga"*. Data updates are carried out periodically and every time there is a change in conditions. The reported health conditions of residents include maternal and child health data as well as degenerative diseases alongside their complications. Second, the Great Surabaya Cadres work together with the Community Health Center in the integrated development place (In Indonesian, *Posbindu*) activities, integrated service place for toddlers (In Indonesian, *Posyandu balita*), and integrated service place for the elderly (In Indonesian, *Posyandu lansia*). In *"Posbindu"* activities, the Great Surabaya Cadres work together with health workers from the Community Health Center to conduct monitoring and early detection of non-communicable diseases which include measuring waist circumference, weight, blood pressure, blood sugar, blood fat levels, and also counseling. In the *"Posyandu balita"* activities, the Great Surabaya Cadres together with midwives monitor children's health, especially toddlers. These activities include body weight, body length/height, head circumference, immunization, provision of additional food and counseling that are routinely carried out once a month. In *"Posyandu lansia"* activities, cadres work together with health workers from the Community Health Center to provide health services to elderly residents. The health services provided include measuring Body Mass Index (BMI), checking blood pressure, blood sugar, cholesterol and health education, as well as simple physical activities such as elderly gymnastics. The third task of the Great Surabaya Cadres is to implement the *"Jumantik"* program. This program is a program to prevent dengue haemorrhagic fever through 3M activities (In Indonesian, 3M = *menguras, menutup, mendaurulang;* in English = drain, close and recycle) [4]. In this program, the great Surabaya cadres will check and ensure that the residential environment is free from mosquito larvae that can cause dengue haemorrhagic fever [5,6].

Monitoring the health conditions of the entire community in a metropolitan city like Surabaya is not an easy thing. The existence of the Great Surabaya Cadres formed by the Surabaya city government is very useful as an extension of the Community Health Center. The involvement of the Great Surabaya Cadres also supports the achievement of health programs initiated by the government [7]. The commitment and cooperation between the Great Surabaya Cadres and health workers at the Community Health Center are expected to be able to improve the health of the Surabaya community as a whole.
